# Diagnostic and Prognostic Implications of FGFR3, TP53 Mutation and Urinary Biomarkers in Urothelial Carcinoma in Pakistani Cohort

**DOI:** 10.3390/jcm14238526

**Published:** 2025-12-01

**Authors:** Muhammad Asif, Faiza Abdul Rashid, Saima Shakil Malik, Dilshad Ahmed Khan, Muhammad Tanveer Sajid, Asma Gul, Muhammad Tahir Khadim

**Affiliations:** 1 Department of Histopathology, Armed Forces Institute of Pathology, National University of Medical Sciences, Rawalpindi 46000, Pakistan; asifwahab2012@gmail.com; 2 Department of Biology, Faculty of Sciences, Allama Iqbal Open University, Islamabad 44000, Pakistan; faiza.rashid@adjunct.aiou.edu.pk; 3 Cardiovascular Research Institute, Baylors College of Medicine, Houston, TX 77030, USA; saima.shakil@bcm.edu; 4 Department of Pathology, National University of Medical Sciences, Rawalpindi 46000, Pakistan; dakhan@cpsp.edu.pk; 5 Armed Forces Institute of Urology, National University of Medical Sciences, Rawalpindi 46000, Pakistan; muhammadtanveersajid@gmail.com; 6 Precisionsmedicinskt Laboratorium, Universitetssjukhuset, 58185 Linköping, Sweden; asma.gul@regionostergotland.se

**Keywords:** urothelial carcinoma, FGFR3, TP53, immunohistochemistry, NMIBC, MIBC, urinary biomarker, Xpert BCM, urine cytology, cystoscopy, genotyping

## Abstract

**Background:** Urothelial carcinoma (UC) presents with clinically heterogeneous disease. There is an emerging need to explore the prognosis of non-muscle-invasive bladder cancer (NMIBC) and muscle-invasive bladder cancer (MIBC). Therefore, we aimed to explore the prognostic value of FGFR3 and TP53 mutations and protein expression and to investigate the diagnostic utility of urine cytology and Xpert bladder cancer monitor (BCM) assay in UC. **Materials and Methods:** A prospective cross-sectional study was conducted in a cohort of 73 Pakistani patients. Cystoscopy, biopsy, tissue diagnosis, UC grade, and stage followed by immunohistochemistry (IHC) and genotyping were recorded. Voided urine samples were also collected for urine cytology and Xpert BCM. Statistical analysis was performed using SPSS version 26.0. A *p*-value ≤ 0.05 was considered statistically significant. **Results:** In our selected patients, the majority were males who had smoking history and the common symptom was hematuria. Our findings suggest FGFR3 IHC expression is strongly linked to low-grade NMBIC (*p* ≤ 0.01). p53 IHC expression supports the findings of the UC grade (*p* ≤ 0.01). A highly significant association (*p* < 0.001) was observed between FGFR3 protein expression and underlying mutations. Pro72Arg polymorphism (*p* = 0.04) was found to be significantly correlated with p53 IHC findings. While comparing cystoscopy with cytology and Xpert BCM, the sensitivity was found to be 85.7% and 58.5%, respectively. **Conclusions:** The integrated approach of IHC with genotyping could improve risk stratification and guide personalized management strategies. Moreover, as cytology is less sensitive to diagnose UC, especially low-grade tumours, Xpert BCM can be used as a promising diagnostic test for both primary and recurrent BC settings.

## 1. Introduction

Bladder cancer (BC) is the tenth most common cancer worldwide, and the incidence is gradually increasing in the developing world [1]. In Pakistan, it is among the top ten cancers, representing 4.9% in males [2]. Tobacco smoking, along with exposure to harmful occupational and environmental carcinogens, is associated with approximately half of BC cases [3,4]. Although less common, familial predisposition still plays a significant role in the development of bladder cancer [5].

Bladder cancer occurs in two main clinical types, including non-muscle-invasive bladder cancer (NMIBC) and muscle-invasive bladder cancer (MIBC). NMIBC accounts for the majority of the newly diagnosed BC cases and is typically restricted to urothelial mucosa and submucosa. It is characterized by frequent recurrences but normally has a favourable prognosis. Conversely, MIBC invades the muscularis propria layer of the bladder and is associated with a higher stage disease with an increased risk of metastasis, poor clinical outcomes, and reduced survival rates. NMIBC and MIBC are characterized by distinct molecular profiles with FGFR3 (fibroblast growth factor receptor 3) and occur in approximately 70% of NMIBC and 15% of MIBC cases. FGFR3 is a member of the receptor tyrosine kinase family and plays an important role in activating pathways that control numerous cellular functions, such as migration, migration, and differentiation. Many of the FGFR3 mutations happens at three hotspots in exons 7, 10, and 15, while 88% of these mutations occur at exons 7 and 10. Most of these mutation are the missense type [6]. Activating mutations in FGFR3 promotes uncontrolled cell proliferation through ligand-independent receptor dimerization and subsequent activation of the downstream signalling pathways, most importantly the RAS-MAPK pathway [7,8].

On the other hand, tumour protein p53 (TP53) is a tumour suppressor gene that encodes a central tumour suppressor protein which regulates cell-cycle checkpoints and apoptosis. Mutations in TP53 gene are more frequent in MIBC and is involved in genomic instability and tumour aggressiveness and corresponds to worse overall survival in UC [9,10,11,12].

The heterogeneity of FGFR alterations demands advanced molecular diagnostics to guide targeted therapies. The US FDA has recently approved FGFR inhibitors, such as erdafitinib, as effective therapeutic options for FGFR3 altered urothelial carcinoma (UC) [13,14]. Since then, emerging data has demonstrated the efficacy of combining erdafitinib with immunotherapy in treating FGFR-altered UC [15].

Moreover, treatments targeting TP53 mutations are presently under clinical research, and their prognostic and therapeutic importance is extensively established. Emerging evidence shows that alterations in the TP53 pathway have a substantial role in modulating immune checkpoint activity, thus shaping the tumour microenvironment and thereby inducing cancer immune evasion and treatment responses and indicating the potential for combined therapeutic strategies [16,17,18].

Cystoscopy remains the gold standard for diagnosing both NMIBC and MIBC. Although it is an invasive method that requires anaesthesia and carries certain risks of complications, it is highly sensitive and accurate. In recent years, several other non-invasive methods have been developed, including AI-based urine tests (such as Toby’s test), bioinformatic approaches, UroVysionassays, and the use of synthetic biomarkers and metabolites for BC detection [19,20].

Recently, newly developed a urine-based non-invasive biomarker assay known as Xpert Bladder Cancer Monitor (BCM) (Xpert, Cepheid, Sunnyvale, CA, USA) has become the hotspot among researchers. This urine-based test quantitates the expression of five micro ribonucleic acids (miRNAs), ABL1, CRH, IGF2, UPK1B, and ANXA10, frequently overexpressed in bladder cancer [21]. It is a feasible test with improved sensitivity and negative predictive value (NPV) as compared to cystoscopy and cytology. This test shows promise as a tool for monitoring disease recurrence and progression in UC patients [22].

Despite ongoing efforts to improve bladder cancer management in Pakistan, significant gaps exist in early diagnosis, standardized treatment protocols, and multidisciplinary care. Moreover, limited access to advanced diagnostics, variable preoperative therapeutic inventions, and low or noncompliance to treatment contribute to frequent advanced stage disease presentations and poorer outcomes [23].

While environmental and infectious risk factors such as cigarette smoking and human papilloma virus (HPV) are acknowledged, their precise role in bladder cancer etiology within the Pakistani population remain underexplored [24,25]. Moreover, prior studies lack comprehensive molecular profiling of key mutations like FGFR3 and TP53 specifically in this demographic population. Therefore, this study is critical to fill these knowledge gaps by evaluating the expressional analysis and clinical relevance of the FGFR3 and TP53 genes in Pakistani UC patients with the aim of advancing targeted therapies and personalized treatment strategies.

## 2. Materials and Methods

### 2.1. Study Design and Ethical Approval

This was a prospective cross-sectional study conducted within a Pakistan cohort, designed to evaluate the diagnostic accuracy of urinary biomarkers assay, Xpert Bladder Cancer Monitor (BCM), and prognostic implications of FGFR3 and TP53 mutations in UC. The research was conducted at the Armed Forces Institute of Pathology (AFIP), Rawalpindi, in collaboration with the Armed Forces Institute of Urology (AFIU)/National University of Medical Sciences (NUMS), Rawalpindi, Pakistan. Ethical approval was granted by the Institutional Review Board of AFIP/NUMS on 26 April 2019 (protocol number Cons-HSP-5/READ-IRB/19l412). Written informed consent was obtained from all participants prior to enrollment.

### 2.2. Patient Recruitment, Clinical Evaluation, and Clinicopathological Data Collection

A total of 150 patients presenting with urinary symptoms, like painless hematuria, frequency, dysuria, and urinary retention, were recruited during the period from 1 March 2022 to 29 February 2024 at AFIP/AFIU/NUMS, Rawalpindi (Figure S1). This cohort also included patients with a prior history of UC. All participants underwent a comprehensive clinical evaluation that included medical history such as presenting complaints, past medical and surgical history, and demographic profiling such as age, gender, smoking status, and family history. The diagnostic work-up of the patients in our study was conducted in accordance with the European Association of Urology (EAU) Guidelines and the NCCN Bladder Cancer Guidelines (2025) [26,27].

### 2.3. Patient Selection Criteria

Patients of any age or gender with evidence of bladder mass identified on ultrasonography (USG) were further evaluated by cystoscopy except those who had a prior history of bladder cancer. In that case they were subjected to cystoscopic examination. All ofthese patients underwent a transurethral resection of bladder tumour (TURBT), and tissue specimens were collected for histopathological examination, immunohistochemistry (IHC), and molecular genotyping. In addition, voided urine samples were also obtained from each patient for cytological examination and Xpert BCM whereas those with benign urological conditions such as active urinary tract infection, urolithiasis, and prostatic pathology were excluded. Moreover, insufficient necrotic tissue samples or those who were unwilling or unable to provide written informed consent were also excluded.

### 2.4. Sample Collection and Processing

#### 2.4.1. Urine and Bladder Tissue Samples

Urine samples (25 mL) collected from all patients prior to TURBT were immediately sent for cytological evaluation. An additional 4.5 mL aliquot of urine was also prepared for analysis using the Xpert BCM (Cepheid, Sunnyvale, CA, USA). Tissue specimens obtained during TURBT were fixed in 10% buffered formaldehyde for 24 h.

#### 2.4.2. Cytopathological Examination

Voided urine samples obtained from study participants were transferred to the cytopathology laboratory for processing. From each specimen, both air dried and wet fixed smears were prepared, and wet fixed smears were promptly fixed in 100% ethanol to ensure cellular preservation. Two staining protocols were applied: Diff-Quik staining on air dried smears for rapid assessment of cellular morphology and Papanicolaou stain on wet fixed smears for detailed cytological evaluation. All slides were examined and reported by two independent cytopathologists in accordance with the Paris system for reporting urinary cytology [28], which provides a standardized framework for categorizing urothelial cells.

#### 2.4.3. Histopathological Examination of Tissue Specimens

FFPE tissue blocks were prepared in an automated tissue processor (Tissue-Tek-VIP^®^6, Sakura, Tokyo, Japan), Japan according to standardized laboratory protocols. Tissue sections of 5 μm thickness were obtained using an automated tissue microtome (Tissue-Tek AutoSection^®^, Sakura). Tissue was mounted on glass slides for subsequent staining and evaluation [29,30]. A histopathological assessment of the tumour grade and stage was performed using the WHO classification guidelines [31].

#### 2.4.4. Hematoxylin and Eosin (H&E) Staining Protocol

Tissue sections were deparaffinized in two successive baths of histoclear and subsequently rehydrated through a graded series of ethanol (100%, 95%, 80%, and 70%) before being rinsed in distilled water. Nuclear staining was performed with Harris hematoxylin, followed by differentiation in 1% acid alcohol and bluing in Scott’s tap water substitute to enhance nuclear detail. Counterstaining of the cytoplasm and extracellular matrix was achieved with Eosin Y. Finally, sections were dehydrated in ascending ethanol concentration, cleared in histoclear, and permanently mounted with DPX (Distyrene Plasticizer Xylene) mounting medium. This standardized procedure yielded optimal nuclear cytoplasmic contrast, allowing reliable histopathological evaluation [30,32].

#### 2.4.5. Immunohistochemistry Using FGFR3 and p53 Antibodies

IHC was performed on the FFPE tissue section of all 73 cases. Slides were deparaffinized in histoclear and rehydrated with serial ethanol dilutions. For both FGFR3 and p53, heat induced antigen retrieval was performed by incubating slides at 95–100°C in a hot water bath using a citrate buffer with apH of 6 for 30 min. For FGFR3, ready to use, pre-diluted FGFR3 antibody (BSB-150, MMAb, Catalogue no: 3724-15) was used. For p53, TP53 Novocastra™ Liquid Mouse Monoclonal Antibody p53 Protein (DO-7) (Leica Biosystems, Nussloch, Germany, Catalogue no: PA0057) at 1:800 dilution was used. After washing, the slides were incubated with diluted biotinylated secondary antibodies at room temperature, followed by colour development using a 3,3′-diaminobenzidine (DAB) solution for 5 min. Lastly, counterstaining with hematoxylin was performed. FGFR3 staining intensity was assessed semi-quantitatively as weak, moderate, or intense, whereas p53 expression was categorized as mutant (positive) or wild type (negative) based on nuclear immune-reactivity patterns. Regarding the antibody controls, high-grade UC was used as positive control for p53, and normal testicular tissue was used as positive control for FGFR3 as recommended by the manufacturer. Background stromal tissue was used as negative control for both. All slides were independently reviewed by two histopathologists for confirmation of findings [33].

### 2.5. Genotyping of FGFR3 and TP53

#### 2.5.1. Genomic DNA Extraction

FFPE tissue blocks from 70 patients were sectioned at 5 µm thickness on a semi-automated rotary microtome (Tissue-Tek AutoSection^®^, Sakura, Japan), and 5–8 sections per case were collected into sterile, low-bind tubes. Paraffin was removed with QIAGEN deparaffinization solution (QIAGEN, Hilden, Germany; Cat. no.: 19093), followed by genomic DNA extraction using the QIAamp DNA FFPE Tissue Kit (QIAGEN, Hilden, Germany; Cat. no.:56404) according to the manufacturer’s protocol. Patients with carcinoma in situ (CIS) (*n* = 3) were not processed owing to insufficient tumour tissue and DNA quality. The FFPE DNA concentration was measured on a Qubit 3.0 Fluorometer (Thermo Fisher Scientific, Waltham, MA, USA) using the Qubit dsDNA High-Sensitivity (HS) Assay Kit (Thermo Fisher Scientific/Invitrogen, Cat. no.: Q32854). DNA purity (A260/A280) was assessed on a NanoDrop 2000 spectrophotometer (Thermo Fisher Scientific, Waltham, MA, USA).

#### 2.5.2. Primer Design and Target Regions

Exon sequences for FGFR3 (exon 7) and TP53 (exon 4) were retrieved from the Ensembl Genome Browser (release 105; human reference genome GRCh38; EMBL-EBI/Wellcome Sanger Institute, Hinxton, UK). Primers were designed in Primer3Plus (web tool) (Table S1) and evaluated in OligoAnalyzer (Integrated DNA Technologies, IDT, Coralville, IA, USA) for secondary structure propensity (hairpins, self-/hetero-dimerization), GC content, T_m compatibility, and predicted off-target complementarity.

Targets included FGFR3 hotspot mutations c.742C>T (p.Arg248Cys, rs121913482, exon 7), c.746C>G (p.Ser249Cys, rs121913483, exon 7), c.882T>C (p.Asn294Asn, exon 7), and the TP53 variant c.215C>G (p.Pro72Arg, rs1042522, exon 4). Variant notation follows the Human Genome Variation Society(HGVS) recommendations relative to canonical RefSeq transcripts (FGFR3 NM_000142; TP53 NM_000546).

#### 2.5.3. PCR Amplification of FGFR3 and TP53

PCR was performed in a 25 µL PCR reaction mixture which contained template DNA, 10 pmol of each primer, 200 μM dNTPs, 1.5 mM MgCl_2_, 1× PCR buffer, and 1 U of Taq DNA polymerase. Amplification was carried out using a thermal cycler under the following cycling conditions. Initial denaturation at 95°C for 5 min was followed by 30 cycles of denaturation at 95 °C for 30 s, annealing at 58°C for 30 s, and extension at 72 °C for 45 s. Final extension was carried out at 72 °C for 5 min [34].

#### 2.5.4. Gel Electrophoresis and Visualization

PCR amplicons were separated by electrophoresis on 1% agarose gels stained with Cybersafe (Thermo Fisher Scientific). Band sizes were compared against a molecular weight marker, and gels were visualized under UV light using a gel documentation system.

#### 2.5.5. DNA Sequencing

PCR amplicons were sent to Macrogen, Inc. for Sanger sequencing, and results were analyzed to identify FGFR3 and TP53 mutations.

#### 2.5.6. Custom PCR and Sanger Sequencing

Genomic DNA extracted from FFPE tissue (Section 2.4.1) together with the study’s target primer pairs for FGFR3 (exon 7) and TP53 (exon 4) (Table S1) were shipped to Macrogen, Inc. (Seoul, Republic of Korea). The provider performed custom PCR amplification and bidirectional Sanger sequencing of the specified targets according to their validated workflow. The provider returned raw chromatogram files (.ab1) and corresponding sequence calls for all samples.

#### 2.5.7. Mutational Analysis

Raw.ab1 files were inspected and trimmed for low-quality ends in BioEdit (v. 7.7.1.0). Forward and reverse reads were assembled, and consensus sequences were aligned to the human reference genome (GRCh38) using the canonical transcripts FGFR3 (NM_000142) and TP53 (NM_000546) as references. Chromatograms were examined for single vs. double peaks at the predefined hotspot positions: FGFR3 c.742C>T (p.Arg248Cys), c.746C>G (p.Ser249Cys), c.882T>C (p.Asn294), and TP53 c.215C>G (p.Pro72Arg). Putative heterozygous calls required a reproducible secondary peak at the same position in both directions with appropriate spacing and baseline separation; ambiguous positions were re-checked manually on the raw traces. All variants were described in HGVS nomenclature relative to the reference transcripts above.

### 2.6. Xpert Bladder Cancer Monitor (BCM) Assay

From all 73 patients, irrespective of a cystoscopically confirmed lesion, urine samples were further analyzed using theXpert BCM (Cepheid, Sunnyvale, CA, USA) according to the manufacturer’s protocol. Within 30 min of collection, a 4.5 mL aliquot of urine was transferred into the assay’s preservative container to stabilize the nucleic acids. Samples were stored at 2–8 °C and processed within 7 days of collection [35].Each preserved specimen was loaded into a single-use Xpert BCM cartridge and analyzed on the Cepheid GeneXpert system. For each sample, a linear discriminant analysis (LDA) score was automatically generated by the system. This score represents the probability of bladder cancer based on a weighted combination of biomarker signals. Results were interpreted using two thresholds: a primary cut off (LDA ≥ 0.22), which has been reported to yield an estimated sensitivity of 0.90–0.95 with moderate specificity [21], and a secondary cut off (LDA > 0.45), corresponding to the manufacturer’s recommended standard for test positivity.

### 2.7. Statistical Analysis

Data are presented as the mean ± standard deviation (SD) for continuous variables. Categorical variables, including age, gender, tumour grade, stage, and smoking status, were compared using the χ^2^ test. Two-way Anova and regression analysis were performed to evaluate the associations between FGFR3 and TP53 mutation status, clinicopathological features, immunohistochemistry, and cystoscopy. A *p*-value ≤ 0.05 was considered statistically significant. All analyses were performed using SPSS version 26.0 (IBM Corp., Armonk, NY, USA) whereas figures were generated with GraphPad Prism 10.

## 3. Results

### 3.1. Clinicopathological Characteristics of Study Patients

This study analyzed the clinicopathological characteristics of 73 UC patients as per the selection criteria. The cohort was predominantly composed of males (85%) with only (15%) females. The mean age of the participants at diagnosis was 63 years, with a range of 21 to 90 years. Smoking history was reported among 81% of patients, while 19% were non-smokers. Moreover, 8% of patients had a family history of cancer, whereas 92% reported no such history. Hematuria, a common symptom in bladder cancer patients, was present in 88% of the patients. Symptoms of dysuria and urgency were found in 64% patients, while 36% did not exhibit these symptoms. Urine cytology was positive in 33% of patients.

Additionally, TNM staging revealed that 4% (*n* = 3) of patients were at pTis, 33% at the pTa stage, 37% at pT1, 25% at pT2, and only 1% at pT3 stage. Most cases (77%) were primary tumours, while 23% of the cases were recurrences. Among the cohort, 77% of patients presented with a primary tumour without a prior history of cancer, whereas 23% of patients presented with tumour recurrences. (Table 1)

### 3.2. Cystoscopic Analysis of Patients

Cystoscopy was performed on all 73 patients to allow the direct visualization of the bladder for the detection of abnormal growth and any abnormal lesions. A total of 96% of patients showed a visible mass, while 4% had negative results with no cystoscopically detectable growth. Patients with invisible growth on cystoscopy had a history of bladder cancer. Cystoscopy was followed by a biopsy and histopathological analysis.

### 3.3. Immunohistochemical Scoring for FGFR3 and p53

**Figure 1:**
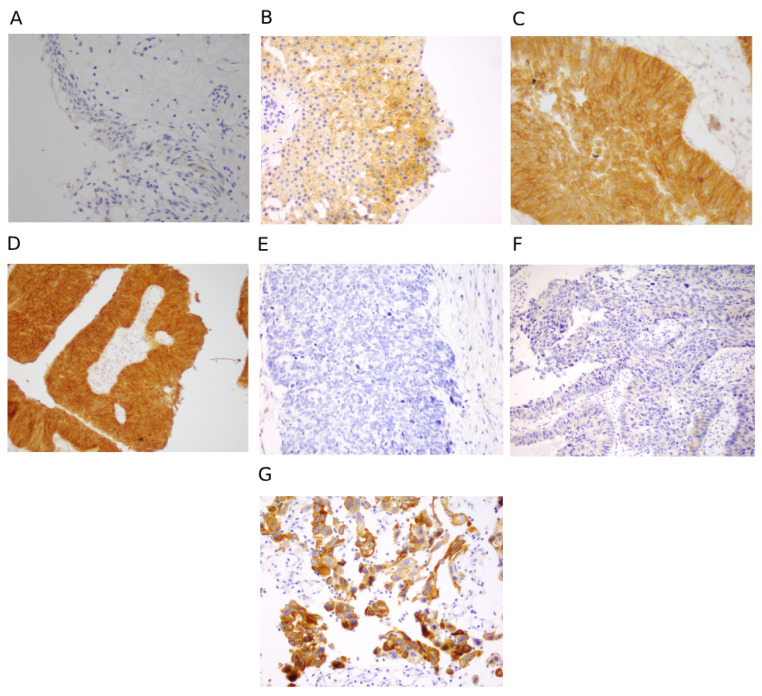
Immunohistochemical expression of FGFR3 antibody. (**A**) FGFR3 stained normal bladder mucosa showing occasional cytoplasmic staining in a few cells but, overall, most of the urothelial cells remain negative. (**B**) FGFR3 stained low-grade UC showing a weak cytoplasmic and membranous staining. (**C**) FGFR3 stained low-grade UC showing moderate cytoplasmic and membranous staining. (**D**) FGFR3 stained low-grade UC showing strong cytoplasmic and membranous staining. (**E**) FGFR3 stained high-grade UC appears to be completely negative. (**F**) FGFR3 stained high-grade UC showing occasional cytoplasmic staining in a few cells but most of the urothelial cells remain negative. (**G**) FGFR3 stained high-grade UC showing strong cytoplasmic and membranous staining. (**B**–**G**): background stroma serves as negative control).

H&E-stained sections of normal urinary bladder mucosa along with CIS and low- and high-grade UC are shown in Supplementary Figure S2. FGFR3 and p53 IHC were performed on all 73 samples. In this study, all types of UC (CIS, NMIBC, and MIBC) demonstrated varied degrees of cytoplasmic as well as membranous expression for FGFR3.Among which, 45, 19, and 9 cases showed weak, moderate, and strong FGFR3 staining, respectively (Figure 1 and Table 2). We have observed that among the 45 weakly stained UC cases, 37 were high-grade, 6 were low-grade, and only two were CIS cases. Out of 19 moderately stained FGFR3 tumours, 8 were high-grade, with 11 being low-grade ones and zero positivity in CIS cases. In particular, of the nine tumours with strong FGFR3 positivity, six were low-grade, two were high-grade, and one was CIS (Table 2). Our findings are consistent with prior evidence that FGFR3 overexpression is strongly linked to low-grade NMIBC, supporting its role as a potential biomarker for favourable prognosis and as a therapeutic target for FGFR-directed therapies.

**Figure 2:**
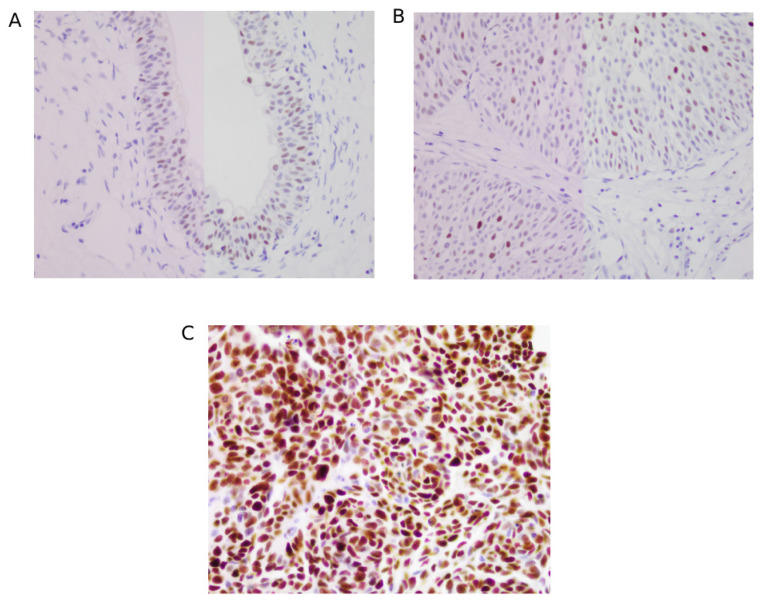
p53 immunohistochemical expression. (**A**) p53 stained section of normal bladder mucosa showing nuclear staining in basal cells with negative staining in the overlying epithelial layer and stromal cells. (**B**) p53 stained low-grade UC showing weak and focal staining in tumour cells. Majority of cells are negative. (**C**) p53 stained high-grade UC showing strong and diffused nuclear staining in most tumour cells. (Background stromal cells are negative and serve as internal negative control).

In this study, out of 52 mutant p53 tumours, 45 were high-grade, 4 were low-grade, and 3 were CIS cases. Figure 2 describes patterns of p53 staining in normal bladder mucosa (A), low-grade UC (B), and high-grade UC (C). In contrast, 19 of the low-grade tumours showed negative/wild type p53 expression, demonstrating that p53 overexpression supports the findings of the histological tumour grade (Table 2).

### 3.4. Mutation Analysis of FGFR3 and TP53

**Figure 3:**
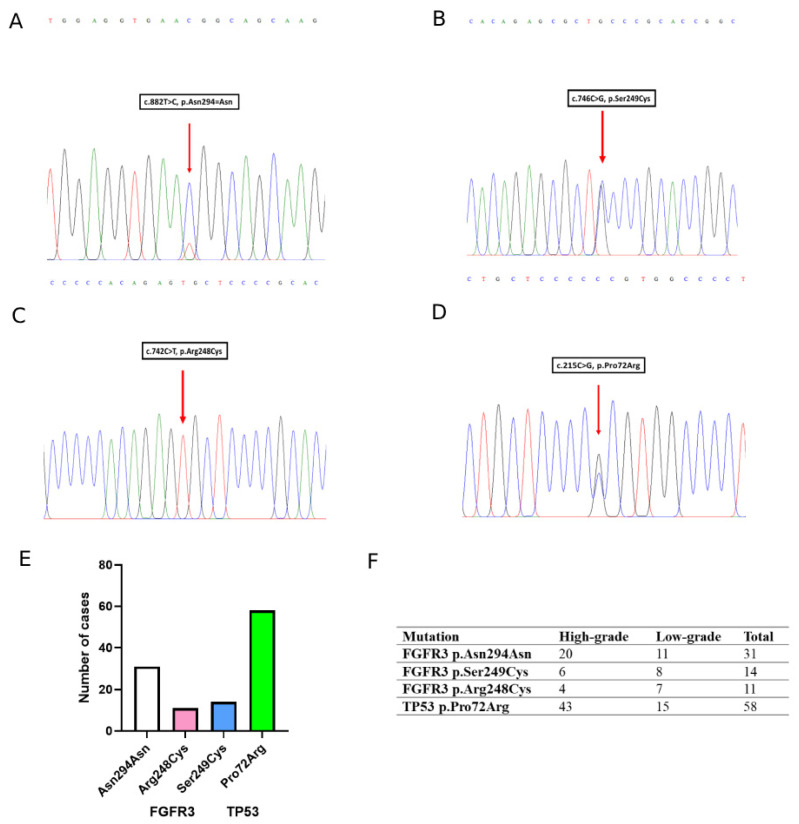
FGFR3 and TP53 mutation profiling in urothelial carcinoma. (**A**–**D**) Representative Sanger sequencing chromatograms showing identified mutations: (**A**) FGFR3 c.882T>C (p.Asn294Asn), (**B**) FGFR3 c.746C>G (p.Ser249Cys), (**C**) FGFR3 c.742C>T (p.Arg248Cys), and (**D**) TP53 c.215C>G (p.Pro72Arg). Arrows indicate the mutation sites. (**E**) Distribution of FGFR3 and TP53 mutations. (**F**) Summary table of mutation frequencies stratified by high-grade and low-grade UC.

PCR amplification and Sanger sequencing were successfully performed on the selected low- and high-grade UC cases to detect FGFR3 and TP53 mutations. The most frequent FGFR3 variant found was the synonymous p.Asn294Asn (c.882T>C) polymorphism, detected in 31 cases. Two pathogenic mutations including p.Ser249Cys (c.746C>G) and p.Arg248Cys (c.742C>T) were found in 14 and 11 cases, respectively. The TP53 p.Pro72Arg (c.215C>G) mutation was found in 58 cases, while 12 cases carried the wildtype allele (Figure 3A–F).

### 3.5. Correlation of FGFR3 Mutations with Immunohistochemical Protein Expression

**Figure 4:**
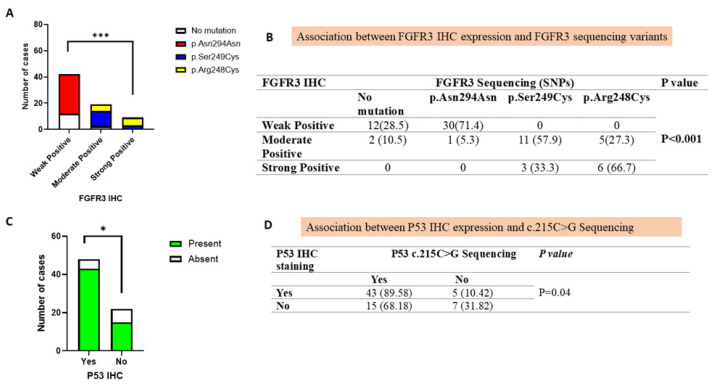
Diagnostic performance of PCR (sequencing) compared with immunohistochemistry. (**A**) A stacked bar chart representing association between FGFR3 polymorphisms and FGFR3 immunohistochemical expression. (**B**) Association between FGFR3 immunohistochemistry and its sequencing variants. (**C**) A stacked bar chart representing association between TP53 c.215›G polymorphism (Pro72Arg) and its immunohistochemical expression. (**D**) Association between p53 IHC expression and c.215C>G Sequencing. (* represents *p* = 0.05; *** represents *p* ≤ 0.0001).

A highly significant association (*p* <0.001) was observed between FGFR3 protein expression and FGFR3 mutational status (Figure 4A,B). We have observed that weak FGFR3 staining was predominantly associated with the synonymous Asn294Asn variant (71.4%), suggesting that weak expression may often represent non-pathogenic or low-impact polymorphism. Whereas moderate FGFR3 staining showed strong correlation with both pathogenic mutations Ser249Cys (57.9%) and Arg248Cys (27.3%), indicating that moderate expression is a marker of functional FGFR3 alterations commonly linked with UC progression. Interestingly, strong FGFR3 staining was exclusively associated with pathogenic mutations Ser249Cys (33.3%) and Arg248Cys (66.7%), emphasizing that high protein expression on IHC correlates with the presence of clinically relevant activating mutations.

### 3.6. Association of TP53 Mutation with Immunohistochemical Protein Expression

In our study, Pro72Arg polymorphism (*p* = 0.04) was found to be significantly correlated with IHC findings. Among cases with mutant p53 IHC staining, 89.6% carried this mutation, whereas among those with negative p53 IHC staining, 68.2% cases had it (Figure 4C,D). These results suggest that the p53 expression observed via IHC strongly correlated with the underlying presence of the Pro72Arg polymorphism, which supports the biological link between mutation-driven dysregulation and protein-level expression.

### 3.7. Xpert Bladder Cancer Monitor (BCM) Analysis

**Figure 5:**
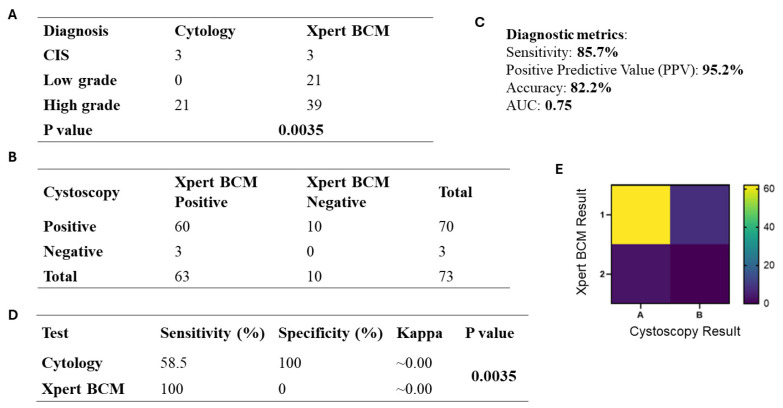
Diagnostic performance of Xpert BCM compared with cystoscopy in UC detection. (**A**) Comparison between diagnostic accuracy of cytology and Xpert BCM as per tumour grade. (**B**) Contingency table comparing Xpert BCM results with cystoscopy findings. (**C**) Diagnostic performance metrics, including sensitivity, specificity, positive predictive value, and overall accuracy. (**D**) Diagnostic metrics and kappa statistics presenting comparison between cytology and Xpert BCM. (**E**) Heatmap representation of concordance between Xpert BCM and cystoscopy results.

The assay was performed on voided urine samples from all selected patients to evaluate its diagnostic accuracy. Overall, 86.3% cases were tested positive, while 13.7% were negative. Xpert BCM performance of UC grades revealed 33% (*n* = 21) low-grade tumours, 62% (*n* = 39) high-grade tumours, while 5% CIS (*n* = 3) cases showed positive results on Xpert BCM (Figure 5A).

### 3.8. Comparison of Xpert BCM with Cystoscopy in UC Patients

In this study, we compared the findings of Xpert BCM and cystoscopy to assess their utility in diagnosing UC (Figure 5B,E). Among the 73 patients, 88.6% were positive on both Xpert BCM and cystoscopy (True Positive), while 11.4% were cystoscopy positive but Xpert BCM negative (False Negative). Among those with negative cystoscopy findings, all (100%) patients tested positive on Xpert BCM (False Positive), while none showed positivity both on cystoscopy and Xpert BCM (True Negative). Xpert BCM with 95.2% PPV represents a positive finding which is highly reliable for the confirmation of UC (Figure 5C). The sensitivity of Xpert BCM was 85.7%, reflecting its ability to correctly identify cases with bladder disease. The overall accuracy (82.2%) of Xpert BCM confirms its effectiveness in diagnosing UC as compared to cystoscopy. The area under curve (AUC) was found to be 0.75, representing its reliability in diagnosing disease.

### 3.9. Comparison of Cytology with Xpert BCM in UC Patients

Figure 5D represents the diagnostic comparison highlighting the strengths and limitations of cytology and Xpert BCM. As known, cytology identified only high-grade tumours showing 100% specificity but a reduced sensitivity of 58.5%,whereas Xpert BCM presented 100% sensitivity as detected for both low- and high-grade tumours. To further confirm the diagnostic performance of Xpert BCM, we tested five normal bladder tissue samples, and all of them turned out to be negative. But we did not use those numbers in the analysis section as this was not a case–control study and was only performed as an internal control. Association between the two tests (cytology and Xpert BCM), based on the tumour grading, turned out to be statistically significant (*p* = 0.0035), whereas kappa tests showed that the two tests are basically detecting varied aspects of tumour biology. These findings suggest that Xpert BCM is highly sensitive to bladder cancer diagnosis, and a combined approach could greatly improve the diagnostic accuracy.

## 4. Discussion

Mutations in the FGFR3 gene serve as prognostic biomarkers and actionable therapeutic targets. Tumours harbouring FGFR3 alterations may benefit from FGFR inhibitors. For example, erdafitinib (Balversa^®^) has been approved for locally advanced or metastatic UC for some of the qualifying FGFR mutations. In resource-limited areas, the execution needs consideration of assay availability, cost, and sequencing strategy [36].

TP53 mutations typically signal a more aggressive phenotype with an increased probability of progression to muscle-invasive disease. As the TP53 gene alone is not currently a targetable alteration, its presence can predict prognosis and treatment intensity. Emerging evidence also links TP53 alterations to the tumour microenvironment and to differential responses to chemotherapy and immunotherapy, underscoring its potential role in multimodal risk stratification [9,10].

This is a preliminary study that provides bases to evaluate the prognostic implications of FGFR3 and TP53 mutations in a Pakistani population. Moreover, we investigated the diagnostic utility of FGFR3 IHC as compared to genotyping. For the first time in our population, this study also provides a comparison between the non-invasive and efficient Xpert BCM assay and the conventional invasive procedures of cystoscopy and cytology.

In the current study, the average age of patients at the time of BC diagnosis was 63 years, like earlier reports [37,38]. Most of the study participants (81%) were smokers [39,40,41] and frequently presented with hematuria and dysuria consistent with the available literature [42,43,44].

We have shown that FGFR3 serves as a marker of early-stage disease whereas mutant p53 expression is associated with tumour aggressiveness, illustrating distinct biological pathways in bladder cancer. We observed moderate to strong FGFR3 IHC positivity in low-grade NMIBC as compared to high-grade MIBC [45]. While considering tumour stage, 95% of NMIBC showed a strong pattern of FGFR3 staining [46,47,48]. However, the majority of low-grade BC revealed wild type P53 staining, a biomarker of high-grade BC consistent with the literature [49].

The current study has explored the utility of FGFR3 and TP53 mutations status and underlying protein expression. We found a strong association between FGFR3 mutation status and protein expression, as evident from the earlier reports [50]. Our study identified that cases with weak positive staining on IHC showed a high rate of synonymous mutation (SM) Asn294Asn. It could not alter the protein expression directly but adapted subtle pathways to produce less concentrated protein expression on IHC. SM is near to pathogenic mutations Ser249Cys and Arg248Cys; therefore, it might be co-inherited with these pathogenic alterations and influence protein expression. Pathogenic splice variant and protein misfolding might also lead to unstable and weak FGFR3 staining [51,52].

UC cases showing moderate to strong staining on IHC carried FGFR3S294C and R248C mutations. It confirms that FGFR3 IHC staining intensity reflects underlying genetic status suggesting strong staining levels, which is highly predictive of oncogenic FGFR3 mutations [53]. In clinical practice, this evidence supports the utility of FGFR3 IHC as a screening tool to identify patients likely to carry FGFR3 mutations. However, sequencing remains the definitive tool for confirmation, particularly for the cases with weak or moderate FGFR3 staining intensity.

The current study showed a strong correlation of FGFR3 mutations with tumour grades. SM was detected in non-invasive high-grade tumours. None of the earlier studies have shown an association of this SM (Asn294Asn) with UC grades. Both high- and low-grade tumours harboured pathogenic FGFR3 S249C or R248C mutations [54]. In contrast, earlier studies reported FGFR3 mutations only in low-grade NMIBC [6,55,56]. This difference showed signs of disease progression and invasiveness. A few studies also showed FGFR3 S294C mutations in high-grade MIBC [56,57,58]. The exact pattern of the mutation status could be cleared in a large set of study patients. These findings suggest FGFR3 mutations have prognostic value and act as predictive biomarker for FDA approved erdafitinib especially designed against FGFR3 altered advanced BC [59].

The Pro72Arg variant of TP53 has been implicated in altering apoptotic potential and influencing cancer susceptibility. Pro72Arg polymorphism is more frequently observed in high-grade tumours as compared to low-grade tumours, which is consistent with previous studies [60,61]. Among p53 mutant IHC cases, the majority carried Pro72Arg polymorphism (TP) while only few showed wild type staining (FN). The false positives (FP) were 68%, and the true negative (TN) were 32%. The reason for FP might be due to unstable or non-accumulation of p53 protein [62]. This finding suggests that p53 expression observed via IHC strongly correlated with the underlying presence of Pro72Arg polymorphism.

In our study, we analyzed the urine-based, non-invasive Xpert BCM assay to measure its diagnostic accuracy and sensitivity as a biomarker of UC. In our study, cystoscopy did not detect three small flat lesions (CIS cases), but Xpert BCM revealed positive results indicative of recurrent bladder disease. This urine-based assay showed 85.7% sensitivity with a PPV of approximately 95%. The results agree with the earlier studies, which showed high sensitivity in the detection of high-grade and overall BC [22,63]. A PPV of 95% depicts its high reliability in detecting positive cases, which contrasts with earlier reports. The Xpert BCM assay has several advantages in terms of clinical practice, such as its rapid turnaround time, non-invasive nature, and standardized automated workflow. These features make it stand out as particularly useful in settings with limited pathological expertise. Most importantly, it helps to reduce the need for frequent cystoscopies, thereby lowering patient burden. However, its clinical utility in resource-limited settings is constrained by the high cost of the platform and cartridges. Still, it is important to keep in mind that it cannot fully replace cystoscopy, specifically in high-risk cases. All of these factors should be taken into consideration while integrating Xpert BCM into routine bladder cancer surveillance [20,21,64].

Furthermore, we correlated the clinical performance of urine cytology and diagnostic accuracy of Xpert BCM. Urine cytology has high specificity but variable sensitivity for high-grade UC. For low-grade UC, it has remarkably lower sensitivity [65,66]. Therefore, urine cytology cannot be recommended as a single test for diagnosing UC but can be used as an additional test [67]. Moreover, cytology has high inter and intra-observer variability in diagnosing UC [68].

### Study Limitations and Future Directions

The present study has certain limitations such as a smaller sample size and their collection from respective centres. Although patients from all over the country visit the selected research centres, collecting samples from all over the country or representative hospitals would create more generalized results. Additionally, the onset of cancer is not the manifestation of a single gene or more and involves a complex interplay of entire genomes and environmental factors. Therefore, many other genes or polymorphisms are also responsible for BC.

Future studies should aim to include larger and more diverse patient populations, ideally through multicenter collaboration across Pakistan, to enhance the generalizability of the findings. Longitudinal studies would be valuable to assess long-term outcomes and disease progression. Further research could explore the underlying biological mechanisms of FGFR3 and p53 alterations, as well as their potential role in guiding personalized therapies. Incorporating advanced genomic, transcriptomic, or proteomic analyses and validating the results in independent cohorts would strengthen the scientific evidence. Additionally, future studies will integrate molecular, protein, and urinary biomarker data with AI-based approaches to improve non-invasive diagnosis and risk prediction in bladder cancer. Longitudinal follow-up and routine BCM assay implementation will support the development of predictive, personalized models [69,70].

## 5. Conclusions

FGFR3 and TP53 mutations describe two recognizable molecular pathways in UC, with FGFR3 mutations prevailing in low-grade, non-invasive tumours and TP53 mutations dominating in high-grade, invasive tumours. The integrated approach of genotyping and IHC could correctly identify these mutations, further helping in risk stratification and guiding both diagnostic and therapeutic decisions. Moreover, immunohistochemistry (IHC) can serve as a cost-effective test in resource-limited settings. Moreover, due to the low sensitivity and specificity of urine cytology, especially for low-grade UC diagnosis, Xpert BCM can serve as an initial, rapid, and non-invasive diagnostic method to enhance the accuracy of bladder cancer diagnosis thus supporting timely diagnostic and management strategies.

## Figures and Tables

**Table 1:** Clinicopathological characteristics of UC patients.

Category	Study Detection	
	(*n*)	(%)
Sex		
Male	62	(85)
Female	11	(15)
Mean age, yr (Range)		
Overall	63	(21–90)
Smoking		
Smoker	59	(81)
Non-Smoker	14	(19)
Family history		
Yes	6	(8)
No	67	(92)
Hematuria		
Yes	64	(88)
No	9	(12)
Dysuria/urgency		
Yes	47	(64)
No	26	(36)
Cystoscopy		
Positive	70	(96)
Negative	3	(4)
Urine cytology		
Positive	24	(33)
Negative	49	(67)
Xpert BCM		
Positive	63	(86.3)
Negative	10	(13.7)
Tumour grade		
Carcinoma in situ	3	(4)
Low-Grade	23	(32)
High-Grade	47	(64)
TNM stage (T stage)		
pTis	3	(4)
pTa	24	(33)
pT1	27	(37)
pT2	18	(25)
pT3	1	(1)
Primary tumour/recurrence		
Primary	56	(77)
Recurrence	17	(23)

BCM: bladder cancer monitor, TNM: tumour node metastasis. pT: primary tumour, UC: urothelial carcinoma, Yr: years.

**Table 2:** Immunohistochemical expression of FGFR3, p53 in UC according to grade and stage (*n* = 73).

Marker	Category	Low n (%)	High n (%)	*p*-Value (Grade)	NMIBC n (%)	MIBC n (%)	*p*-Value (Stage)
FGFR3	Weak	6 (13%)	37 (82%)	0.0008	28 (62%)	17 (38%)	0.012
	Moderate	11 (58%)	8 (42%)		5 (83%)	1 (17%)	
	Strong	6 (67%)	2 (22%)		21 (95%)	1 (5%)	
p53	Mutant+	4 (8%)	45 (87%)	<0.0001	32 (63%)	19 (37%)	0.0024
	Wild–	19 (90%)	2 (10%)		22 (100%)	0 (0%)	

FGFR3: fibroblast growth factor receptor 3, p53: tumour suppressor protein 53, UC: urothelial carcinoma, NMIBC: non-muscle-invasive bladder cancer, MIBC: muscle-invasive bladder cancer.

## Data Availability

The data that support the findings of this study are available from the corresponding author upon reasonable request.

## Supplementary Materials

The following supporting information can be downloaded at: https://www.mdpi.com/article/10.3390/jcm14238526/s1, Figure S1: Flow chart representing patient’s inclusion and exclusion criteria; Figure S2: Histopathological spectrum of normal urothelial mucosa, low grade UC and high grade UC (C); Table S1: Primer’s details used for sequencing

## Abbreviations

The following abbreviations are used in this manuscript:

FGFR3	Fibroblast growth factor receptor-3
TP53	Tumour protein p53
BC	Bladder cancer
UC	Urothelial carcinoma
NMIBC	Non-muscle-invasive bladder cancer
MIBC	Muscle-invasive bladder cancer
IHC	Immunohistochemistry
BCM	Bladder cancer monitor
US FDA	United States Food and Drug Administration
TURBT	Transurethral resection of bladder tumour
FFPE	Formalin fixed paraffin embedded
H&E	Hematoxylin and eosin
µm	Micron metre
DNA	Deoxyribonucleic acid
PPV	Positive predictive value
CIS	Carcinoma in situ
TNM	Tumour node metastasis
HVGS	Human Genome Variation Society
MAPK	Mitogen activated protein kinase
miRNAs	Micro ribonucleic acids
LDA	Linear discriminant analysis
AUC	Area under curve
SD	Standard deviation
CI	Confidence interval
OR	Odds ratio
SPSS	Statistical package for social sciences
