# Supplementary material for: Diagnostic and Prognostic Implications of FGFR3, TP53 Mutation and Urinary Biomarkers in Urothelial Carcinoma in Pakistani Cohort
Source: J Clin Med. 2025 Dec 1;14(23):8526. doi: 10.3390/jcm14238526 (PMC12692803; doi:10.3390/jcm14238526)
Supplement: Supplementary file 1 [file jcm-14-08526-s001.zip › jcm-3957761-supplementary.pdf]

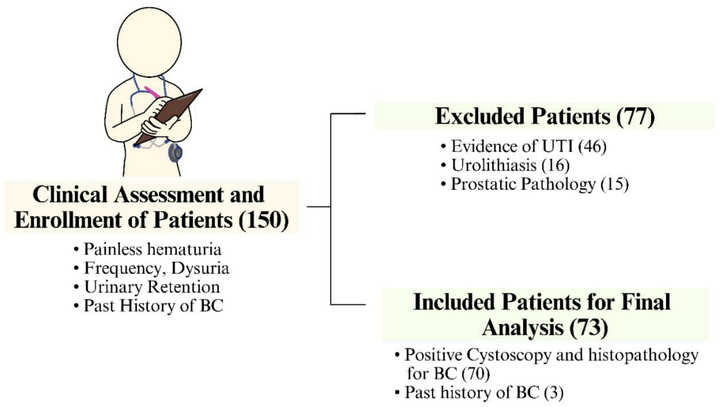

Figure S1: Flow chart representing patients inclusion and exclusion criteria

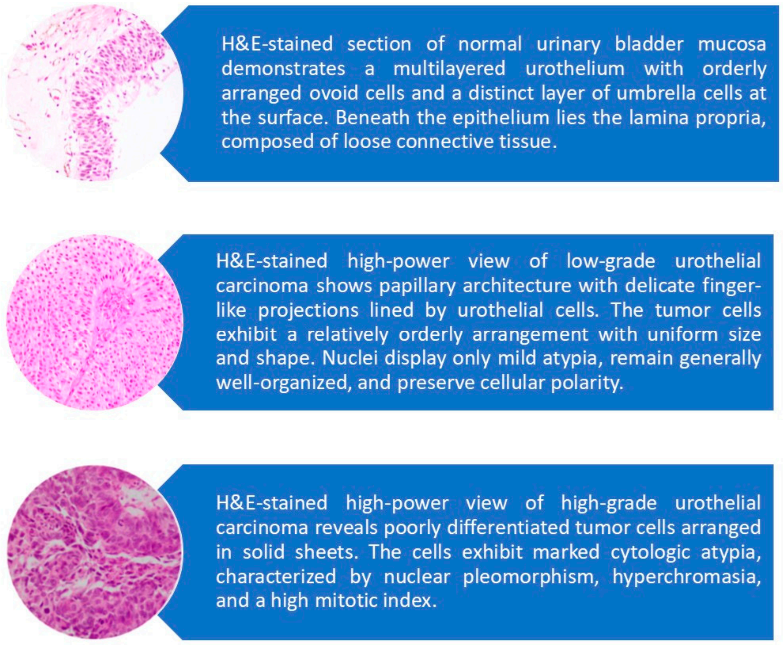

Figure S2: Histopathological spectrum of normal urothelial mucosa, low grade UC and high grade UC (C)

Table S1: Primer's detail used for sequencing

FGFR3	Primer seq (5' to 3')
**Labeling**	**Sequence**
**rs121913482F**	GCGTCGTGGAGAACAAGTTT
**rs121913482R**	GAAGCTCCAACCCCTAGACC
**rs121913485F**	TCACTGGCGTTACTGACTGC
**rs121913485R**	GGTGAGCAGAGACGAGGAGA
**F-TP53-exon4**	TCTGACTGCTCTTTTCACC
**R-TP53-exon4**	ATTGAAGTCTCATGGAAGC
